# Abnormal Neural Connectivity in Schizophrenia and fMRI-Brain-Computer Interface as a Potential Therapeutic Approach

**DOI:** 10.3389/fpsyt.2013.00017

**Published:** 2013-03-22

**Authors:** Sergio Ruiz, Niels Birbaumer, Ranganatha Sitaram

**Affiliations:** ^1^Departamento de Psiquiatria, Centro Interdisciplinario de Neurociencias, Escuela de Medicina, Pontificia Universidad Catolica de ChileSantiago, Chile; ^2^Institute of Medical Psychology and Behavioral Neurobiology, University of TübingenTübingen, Germany; ^3^Ospedale San Camillo, Istituto di Ricovero e Cura a Carattere ScientificoVenezia – Lido, Italy; ^4^Department of Biomedical Engineering, University of FloridaGainesville, FL, USA; ^5^Sri Chitra Tirunal Institute of Medical Sciences and TechnologyThiruvananthapuram, Kerala, India

**Keywords:** schizophrenia, connectivity, fMRI, brain-computer interfaces, brain self-regulation, neurofeedback

## Abstract

Considering that single locations of structural and functional abnormalities are insufficient to explain the diverse psychopathology of schizophrenia, new models have postulated that the impairments associated with the disease arise from a failure to integrate the activity of local and distributed neural circuits: the “abnormal neural connectivity hypothesis.” In the last years, new evidence coming from neuroimaging have supported and expanded this theory. However, despite the increasing evidence that schizophrenia is a disorder of neural connectivity, so far there are no treatments that have shown to produce a significant change in brain connectivity, or that have been specifically designed to alleviate this problem. Brain-Computer Interfaces based on real-time functional Magnetic Resonance Imaging (fMRI-BCI) are novel techniques that have allowed subjects to achieve self-regulation of circumscribed brain regions. In recent studies, experiments with this technology have resulted in new findings suggesting that this methodology could be used to train subjects to enhance brain connectivity, and therefore could potentially be used as a therapeutic tool in mental disorders including schizophrenia. The present article summarizes the findings coming from hemodynamics-based neuroimaging that support the abnormal connectivity hypothesis in schizophrenia, and discusses a new approach that could address this problem.

## Introduction

Schizophrenia is a debilitating and often devastating brain disorder, characterized by a heterogeneous presentation of psychological and behavioral dysfunctions. It is now clear that among the classical description of its symptomatology (delusions, hallucinations, “positive symptoms,” “negative symptoms,” etc.), schizophrenia is characterized by dysfunctions in multiple cognitive domains, which highly correlate with the general functioning of the patient.

In the last decades, and thanks to the development of techniques like neuroimaging, the neural bases of these dysfunctions have started to be elucidated. A number of cognitive impairments in schizophrenia have been specifically linked to disturbances of certain brain areas. For instance, working memory (WM) impairments have been linked to dorsolateral prefrontal cortex (DLPFC) disturbances (Manoach et al., 2000; Perlstein et al., 2001), and facial emotion processing deficits have been associated with limbic abnormalities (Gur et al., 2002; Kosaka et al., 2002).

However, single locations of structural and functional abnormalities are insufficient to explain the diverse psychopathology of the disease. With the development of new methods that can measure the coupled or coordinated action of multiple brain areas, current models postulate that impairments associated with schizophrenia arise from a failure to integrate the activity of local and distributed neural circuits, an idea that can actually be tracked to earliest research in the disease. In fact, more than a century ago Wernicke (1906) posited an abnormal communication between frontal and parietal areas as a key abnormality in schizophrenia. Nowadays, it is postulated that cognitive symptoms of schizophrenia are related to a failure to integrate the activity of local and distributed neural circuits (Andreasen et al., 1998; Phillips and Silverstein, 2003; Gaspar et al., 2008), in the so called “disconnection hypothesis” (Friston and Frith, 1995; Stephan et al., 2006).

Abnormal brain connectivity in schizophrenia has received support from different methodologies. In terms of functional imaging, several studies have shown that abnormal neural connectivity is associated with different cognitive deficits of the disease (please, see Abnormal Brain Connectivity in Schizophrenia. Evidence from Hemodynamics-Based Neuroimaging Techniques). Similarly, abnormal patterns of electrophysiological measures of functional connectivity have also been reported during different cognitive and sensory tasks (Uhlhaas et al., 2008; Hinkley et al., 2011; Uhlhaas, 2012). Neural anatomical disconnectivity has been also suggested using structural MRI techniques such as diffusion tensor imaging (DTI) (Kunimatsu et al., 2012; Nakamura et al., 2012; Roalf et al., 2013).

This article comprises two sections. First, we will show the evidence of abnormal brain connectivity in schizophrenia coming from functional imaging, particularly hemodynamics-based neuroimaging. Secondly, we will explain how Brain-Computer Interfaces based on real-time fMRI (fMRI-BCI) could address this problem.

### Abnormal brain connectivity in schizophrenia. Evidence from hemodynamics-based neuroimaging techniques

Hemodynamic-based brain neuroimaging methods (e.g., positron emission tomography – PET, fMRI) are non-invasive techniques with a spatial resolution that allows more precise understanding of the localized neural substrates than conventional methods based on electrophysiology. Using these techniques, different statistical methods have been developed for the study of connectivity. A useful distinction can be made between methods that measure “functional connectivity” and methods that measure “effective connectivity.” Functional connectivity refers to methodologies that consider only correlations of brain signals across time (Friston, 2011). This approach neither necessarily implies causality (the direction of information flow), nor connection between two brain regions is director or indirect (e.g., through a third region). Despite these limitations, correlation-methods perform with high sensitivity estimating the presence of a network connection with fMRI signals (Smith et al., 2011). Functional connectivity techniques usually include mapping using seed voxel correlations, principal components analysis, and partial least squares methods. Effective connectivity refers to methods that attempt to describe or make inferences about the direction of influence between regions, and include Granger causal mapping, mapping based on psychophysiological interactions (PPI), structural equation modeling (SEM), and multivariate autoregressive modeling (for reviews on methods of functional and effective connectivity with hemodynamic-based neuroimaging techniques, please, see Horwitz, 2003; Rogers et al., 2007).

Currently, a substantial number of PET and fMRI studies have reported a disturbed connectivity in schizophrenia in a wide range of cognitive tasks. In the following lines we will summarize the evidence coming from studies exploring three main cognitive realms altered in schizophrenia: language (semantic processing), WM, and emotional processing.

### Language-related tasks

Language (semantic) abnormalities have been noted in schizophrenia since early conceptualizations of the disease. Functional imaging studies and also volumetric analysis of gray matter have reported alterations in several language-related areas such as inferior frontal gyrus (IFG), superior temporal gyrus (STG), and inferior parietal lobule. Even before neuroimaging methodologies were available to explore brain connectivity, it was hypothesized that abnormal language (and thought) could be due to an abnormal connectivity of language-related brain areas.

In an early study on brain connectivity with a small sample of schizophrenia patients, Spence et al. (2000) tested the fronto-temporal disconnectivity hypothesis exploring regions whose activity correlate with left DLPFC (an area often found dysfunctional in schizophrenia). Although no support for this hypothesis was found, results showed that schizophrenia patients exhibited a relative disconnectivity between left DLPFC and anterior cingulate cortex (ACC) compared to controls while performing a verbal fluency task.

In a subsequent study, Lawrie et al. (2002) used a hypothesis-driven approach to investigate fronto-temporal dysfunctions. The examination of left DLPFC and left middle/superior temporal cortex showed that schizophrenia patients had a reduced correlation coefficient between these areas during a sentence completion task. Interestingly, the deficit in connectivity was correlated with the severity of auditory hallucinations, providing evidence that abnormal coupling of brain areas could be related to cognitive deficits and positive symptomatology in the disease.

Jeong et al. (2009) specifically investigated the connectivity between pars triangularis (IFG) and other brain areas during semantic encoding. The activity of pars triangularis was significantly less correlated with the activity of areas of the left language network in schizophrenia compared to controls. In particular, the mean time-series of the left IFG were less correlated with the left middle temporal gyrus/left superior temporal sulcus, left superior parietal lobule/intraparietal sulcus/supramarginal gyrus, right superior parietal lobule/intraparietal sulcus, left globus pallidus, left thalamus, superior frontal gyrus, precentral gyrus, cerebellum, and right IFG.

These studies included mostly chronic medicated patients, making it difficult to determine whether the findings were due to the disease *per se* or to the chronic use of medication. However, at least two studies have used a different experimental group. Boksman et al. (2005) examined connectivity in first episode, drug-naive schizophrenic patients, using the PPI methodology. PPI is a hypothesis-driven approach that evaluates task-dependent changes in the degree that one region predicts or explains the activity of another (Friston et al., 1997). In this study Boksman et al. (2005) PPI was used to estimate which Blood-oxygen-level dependence (BOLD) response significantly covaried with the maximally activated voxel within the right anterior cingulate while performing a word fluency task. Results reported a widespread unfocussed interaction between the right ACC and other brain areas, without displaying the normal localized activation between anterior cingulate and language areas such as medial left temporal cortex.

In the study conducted by Whalley et al. (2005), a large sample of subjects at high genetic risk of schizophrenia but not having the disease was examined during a sentence completion task. Subjects did not display the reduced prefrontal-temporal connectivity reported by Lawrie et al. (2002) using a similar task in patients with schizophrenia. This might indicate that this particular prefrontal-temporal abnormal connectivity could be associated with the clear manifestation of the disease and might be linked to symptoms of the fully developed psychopathology. Furthermore, a reduced connectivity between prefrontal regions and cerebellum was found in the high genetic risk group, suggesting that this might be a trait-related phenomenon, which could lead to an abnormal coordination of mental processing.

In summary, several studies that have examined language brain circuits suggest abnormal connectivity of fronto-temporal areas in patients with the diagnosis of schizophrenia. The lack of uniformity among the results might arise due to the use of different language tasks, the methodology used for the analysis and the clinical heterogeneity of the participants. Additionally, these studies suggested that abnormalities in brain connectivity might include other brain circuits such as cingulate cortex and cerebellum, and are not just due to the effect of antipsychotic medication.

### Working memory

Working memory, described as the cognitive process that refers to the temporary retention and manipulation of information for a short period of time to solve problems or guide behavior, has been extensively studied in schizophrenia. Abnormal activation of areas involved in WM process has been consistently reported, particularly in the DLPFC.

In an early study, Kim et al. (2003) explored whether schizophrenia patients would display an abnormal connectivity in WM networks, using an N-back sequential task. Examining the brain areas that displayed significant activations related to the task, healthy subjects displayed a correlated activity between lateral prefrontal cortex and bilateral inferior parietal regions. No correlation was found in patients, suggesting that besides a fronto-temporal abnormal connectivity, fronto-parietal coupling could also be disrupted in schizophrenia.

Schlosser et al. (2003) examined schizophrenia patients under a two-back WM task using a measure of effective connectivity, i.e., SEM. SEM is a multivariate linear regression tool that allows the estimation of the directions and strengths of influences between variables, and that needs the pre specification of different models of network connections based on prior hypothesis or knowledge (Penny et al., 2004; Rogers et al., 2007). Exploring a cortical-subcortical-cerebellar network of mnemonic information processing equivalent to the model of “cognitive dysmetria” (Andreasen et al., 1998), Schlösser (Schlosser et al., 2003) found an improper effective connectivity in schizophrenic patients, who had a reduced connectivity within prefrontal-cerebellar projections and an enhanced connectivity in the thalamo-cortical pathways (that might reflect a compensatory strengthening of connections in the presence of disrupted cortical-cerebellar circuitry). This finding is compatible with the results reported by Whalley et al. (2005) in high-risk subjects, providing support to the idea that this prefrontal-cerebellar abnormal connectivity might not be due only to the chronic use of medication.

Meyer-Linderberg and colleagues targeted a different brain area. Considering that multiple abnormalities have been demonstrated in the hippocampal formation in schizophrenia, the authors explored the functional connectivity of this area, using an N-back WM task (Meyer-Lindenberg et al., 2005). Results indicated an abnormal unmodulated persistent functional coupling (fMRI time-series correlations) between DLPFC and hippocampus in schizophrenia patients under WM load.

Henseler et al. (2010) tested whether abnormal connectivity is present in different functional networks supporting independent components of WM: articulatory rehearsal, non-articulatory maintenance of phonological information, and the maintenance of visuospatial information. Using PPI methodology, it was showed that schizophrenia patients display complex patterns of connectivity compared with healthy controls, with reduced connectivity and abnormal negative connectivity among the regions of interests (ROIs). Interestingly, altered prefrontal-hippocampal connectivity during the non-articulatory maintenance of phonological information and parieto-occipital connectivity during the maintenance of visuospatial information were associated with higher positive symptoms, providing a possible explanation for the development of delusions and disorganization symptoms. Although methodologically different, these results replicate the findings of Meyer-Lindenberg et al. (2005), showing again an abnormal WM-related connectivity between fronto-hippocampal areas.

Meda et al. (2009) used a modified version of the Stenberg item recognition paradigm and independent component analysis (ICA) to explore functional connectivity during both encoding and recognition phases of WM. ICA is a statistical method for separating a multivariate signal into additive subcomponents which has found fruitful application in the analysis of fMRI data (McKeown and Sejnowski, 1998). It is a data-driven technique that has the capacity to differentiate different sources of signal (separating artifactual components of the signal from those task-related). The results of the study by Meda et al. (2009) revealed that schizophrenia patients differ from healthy subjects with regard to their weaker functional connectivity, in a network comprising the prefrontal cortex, ACC, medial temporal, basal ganglia, and parietal regions. Again, patients displayed a prefrontal-parietal disconnection compared with healthy subjects.

The above studies examined medicated adult patients. Crossley et al. (2009) examined a group of subjects with prodromal symptomatology “at risk,” and patients who just suffered a first episode of psychosis using dynamic causal modeling (DCM). DCM is a hypothesis-driven methodology in which models are generated, models that estimate the intrinsic connections between sources and changes in the connections that come about through experimental manipulations (Friston et al., 2003; Kiebel et al., 2009). A Bayesian estimation procedure is then used to estimate the best model. In the study by Crossley et al. (2009), DCM was used to specifically explore the connectivity between temporal cortex and other regions during an N-back task. The results showed that neither of the experimental groups displayed the negative coupling between STG and middle frontal gyrus that healthy controls displayed (first episode patients showed a positive coupling, and “at risk” subjects showed almost no coupling). Hence, an abnormal fronto-temporal disconnection could be present even in non-psychotic individuals in prodromal states of the disease.

In summary, studies on WM in schizophrenia have shown abnormal connectivity among different circuits connected to frontal lobes, including fronto-parietal, fronto-cerebellar, and fronto-hippocampal networks. At least one study suggests that a fronto-temporal abnormal connectivity during WM tasks could be present at prodromal states of the disease.

### Emotional processing

Abnormal emotional processing is considered one of the core symptoms of schizophrenia since its earliest descriptions (Bleuler, 1911), encompassing deficits in emotion experience, expression, and recognition (Kohler and Martin, 2006).

In particular, abnormal perception of emotional faces has been extensively reported in schizophrenia, is associated with poor social functioning (Couture et al., 2006) and is known to persist over the course of the illness (Addington and Addington, 1998; Hooker and Park, 2002). Several reports have stated that this deficit is more noticeable for negative emotions (Kohler et al., 2000, 2003; Chambon et al., 2006; Schneider et al., 2006). Among these, abnormal perception of fear faces might be particularly prominent and could contribute to delusions, paranoia, and impaired social interaction. Neuroimaging studies have shown that fronto-limbic and paralimbic areas (particularly amygdala) are associated to this phenomenon in schizophrenia.

Das et al. (2007) examined brain connectivity under conscious and unconscious perception of fearful faces using PPI. During conscious perception of fear faces, healthy subjects and schizophrenia patients showed opposite patterns of connectivity between amygdala and visual cortex, and amygdala and ACC. For non-conscious stimuli (presented below the perceptual threshold), schizophrenia subjects displayed an abnormal negative co-variation between the amygdala and medial prefrontal cortex (MPFC)/ACC. The experiment showed for the first time that patients with schizophrenia have an abnormal pattern of functional connectivity in the “indirect” and “direct” amygdala networks for both conscious and non-conscious perception of fear stimuli. The direct amygdala network is a subcortical pathway that bypasses the visual-striate-cortex, and is reportedly involved in fear processing regardless awareness (Linke et al., 1999). The indirect pathway to the amygdala involves the striatum and lateral geniculate nucleus of thalamus, and is involved in the conscious processing of fear stimuli (Hariri et al., 2003; Das et al., 2005). The authors (Das et al., 2007) suggest that this abnormal connectivity between amygdala and prefrontal areas might contribute to a “schism” between emotion and thought. Furthermore, the abnormal connectivity for unconscious stimuli might suggest impairment in the automatic response to salient stimuli, therefore contributing to misattribution of threat signals.

Fakra et al. (2008) explored brain connectivity in response to emotional faces, using two facial affect recognition conditions: an intuitive emotional condition (“matching” emotional faces) and a cognitive demanding condition (“labeling” emotional faces). A negative correlation between prefrontal areas and left amygdala was found in the labeling condition only in healthy subjects, suggesting that prefrontal cortex provides a top-down modulation of amygdala in a cognitively demanding emotional task. The authors propose that the lack of connectivity between amygdala and prefrontal regions and the increased activation of regions associated with facial feature analysis in schizophrenia might reveal that patients used a cognitive approach when identifying facial affect (feature-based) instead of an holistic and configuration-based approach.

Satterthwaite et al. (2010) examined the interaction between limbic regions involved in emotion processing and parietal regions involved in “recognition memory” in a task in which subjects were asked to recognize neutral faces that were previously presented (“was this face presented before?”). Patients exhibited a different pattern than healthy subjects, with an increased connectivity (correlation) among amygdala and other limbic areas, and an increased connectivity (in a form of a diminished negative correlation) between amygdala and cortical regions. The authors suggested that this increased sensitivity of the amygdala to threatening stimuli might negatively affect the function of cortical regions involved in cognition.

Anticevic et al. (2011) used negative and neutral emotional pictures from the International Affective Picture System (IAPS) (Lang et al., 2008) to modulate a perceptual decision with minimum cognitive load. Although patients and healthy subjects did not differ in behavioral measurements, a weaker amygdala-prefrontal coupling was found in patients, suggesting that amygdala-frontal connectivity dysfunction can arise even in a task with minimum cognitive load, and even when no clear behavioral abnormality is apparent.

Using the same kind of stimuli (IAPS pictures) in a task that involved reappraisal of negative stimuli, subjects prone to psychosis did not show the coupling between amygdala and prefrontal areas that healthy subjects displayed toward negative visual stimuli, a factor that might underlay a reduced cognitive control of emotions and vulnerability to psychosis (Modinos et al., 2010).

In summary, most of the studies that explored functional and effective connectivity targeting amygdala during emotion processing in schizophrenia have found abnormal connectivity between amygdala and prefrontal cortex. Among these, one study suggested that these alterations could not only be part of the full-psychopathology of schizophrenia but also of the vulnerability to psychosis.

### Summary

Accumulating evidence arising from neuroimaging studies supports the idea that schizophrenia is a disorder of improper brain connectivity. Although early studies focused only on the fronto-temporal disconnectivity hypothesis, subsequent studies have moved on to include both reduced and enhanced connectivity among several brain areas, including DLPF with the temporal lobe, parietal lobe, hippocampal formation, thalamus and cerebellum, cingulate cortex, and also limbic regions (particularly amygdala-frontal connections).

Apart from the study of task-related brain regions during task performance, recent studies of brain connectivity have focused on the “resting state” of the brain. This methodology has not only generated much interest in the neurophysiological basis of the resting state brain activity, but also has practical advantages as it does not require an explicit task design (Raichle and Mintun, 2006). Experiments using this methodology have reported dysfunctional integration in widely distributed networks among cortical and subcortical structures in schizophrenia, and have found both reduced and enhanced connectivity between different brain areas (Liang et al., 2006; Zhou et al., 2007). Abnormalities in functional connectivity during resting state have also showed correlations with general psychotic symptomatology (Bluhm et al., 2007) and aggression (Hoptman et al., 2010).

In summary, despite methodological limitations and heterogeneity of the results, ample evidence supports the idea that a failure in the communication and coordination between different brain regions may account for a wide range of symptoms in schizophrenia, including psychosis, emotional disturbances, and cognitive dysfunction, and that at least some of these disturbances can be consider “trait” features, presenting themselves even in early stages of the illness, and not necessarily related to other factors as the use of antipsychotic medication.

Considering all this information, it is natural to wonder whether abnormal connectivity can be modified by external manipulation. In fact, a few reports have suggested that brain connectivity could be modulated with the use of antipsychotics. At the synaptic level, there is information that shows that antipsychotic medication might modulate the presynaptic machinery, therefore modulating the synaptic transmission, and that different mechanisms of action at this level could be partially responsible for different profiles of antipsychotics (either their positive or negative effects), or even the particular efficacy of Clozapine (Barr et al., 2006; Barakauskas et al., 2010). Large-scale network connectivity might also be modulated by the use of medication (Kikuchi et al., 2007; Lui et al., 2010; Sambataro et al., 2010), although the implications of these findings need much further research.

Despite the progress in understanding the neural substrates of schizophrenia, current treatments mainly based on antipsychotics do not incorporate existing know-how in etiology and pathogenesis of the disease, i.e., the abnormal connectivity phenomenon. In fact, to date there are no treatment methods that can produce a direct and significant change in brain connectivity, or are specifically designed to tackle this problem.

We would like to propose a novel technique, namely BCIs based on real-time fMRI, to study and potentially alleviate mental disorders such as schizophrenia, by training patients to self-regulate single brain regions as well as modulate brain network connectivity.

## Brain Connectivity Modulation by Learned Self-Brain Regulation Using fMRI-BCI

### Brain-Computer Interfaces

Brain-Computer Interfaces are novel techniques that utilize neurophysiological brain signals to activate or deactivate external devices or computers (Birbaumer and Cohen, 2007). Recently, the development of BCIs based on metabolic activity of the brain using real-time fMRI (Weiskopf et al., 2007) has become feasible. In contrast to the conventional fMRI that allows analysis of images only after the scan is finished, real-time fMRI allows simultaneous acquisition, analysis, visualization, and feedback of whole brain images. The high spatial and temporal resolution afforded by this method have enabled to train subjects to volitionally control specific brain areas by feedback of the activity of these regions, in a form of a “neurofeedback” based on BOLD signal. Several studies have provided significant evidence that healthy individuals can learn to voluntarily regulate brain activity in different brain areas such as supplementary motor area, STG, parahippocampal place area, ACC, insula, Broca’s area, and amygdala (Yoo and Jolesz, 2002; Posse et al., 2003; Weiskopf et al., 2003, 2004a; deCharms et al., 2004; Yoo et al., 2004; Caria et al., 2007) (for reviews, see Weiskopf et al., 2004b, 2007; Sitaram et al., 2009; Caria et al., 2012). The behavioral change produced by fMRI-BCI can be demonstrated with a few examples: DeCharms showed that with fMRI-BCI, subjects were able to learn control of the activation of rostral ACC, and that this self-regulation induces changes in pain perception (deCharms et al., 2005). Similarly, Rota et al. (2009) used fMRI-BCI to train subjects to enhance BOLD activations in the right IFG, leading to changes in speech processing and modulating language-related performance.

Studies have shown that brain self-regulation with fMRI-BCI can lead to behavioral modifications. Could this change also produce changes in the connections of the brain networks? In order to develop this idea, and to further illustrate the application of fMRI-BCI, we will describe a few experiments conducted in our laboratory.

**Figure 1:**
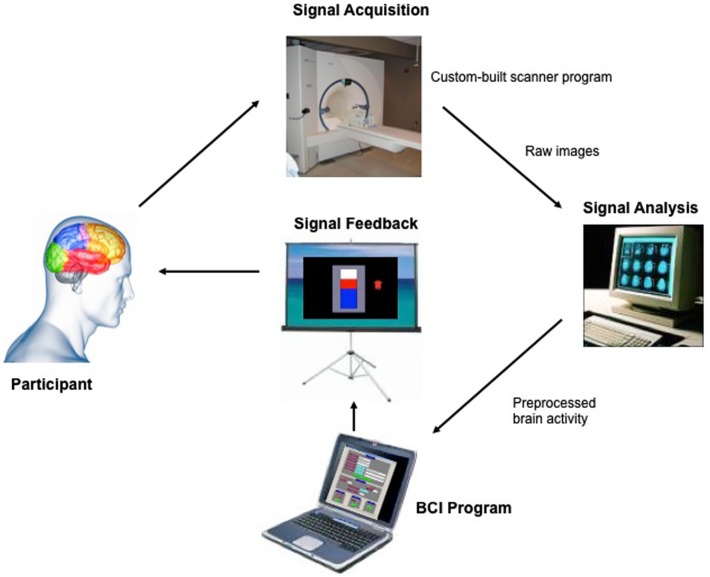
**“Architecture of the fMRI-BCI system.”** The Tübingen fMRI-BCI is implemented as a closed loop system with the following major components: (1) the Participant, (2) Signal Acquisition, (3) Signal Analysis, and (4) Signal Feedback. Localized brain activity is measured by fMRI using the BOLD effect using a 3-T whole body scanner (Trio, Siemens, Erlangen, Germany) with a 12-channel head coil. The signal analysis component is implemented using the Turbo-Brain Voyager (TBV) software (Brain Innovations, Maastricht, the Netherlands) (Goebel, 2001). The signal analysis component retrieves reconstructed images, and performs data preprocessing (including 3D motion correction) and statistical analysis. The average BOLD time-series of selected regions of interest are stored in a continuously updated file, and then exported to the custom-made visualization software which provides feedback to the subject, in real-time reproduced with permission from Sitaram et al. (2007).

Exploring the use of this technique on brain areas of emotion, our group investigated whether healthy subjects can voluntarily gain control over anterior insular using fMRI-BCI (Caria et al., 2007). Insula cortex is an anatomically complex mesocortical structure and part of the paralimbic system that plays a central role in sensory integration, emotion, and cognition (for reviews, see Augustine, 1996; Ture et al., 1999; Craig, 2009). Modulation of the insular activity with fMRI-BCI training might be relevant for different psychiatric diseases such as social phobia, antisocial behavior, schizophrenia, and addictive disorders, in which this area is critically involved (Nagai et al., 2007; Naqvi and Bechara, 2009). In the study by Caria et al. (2007) nine healthy subjects were trained to voluntarily control the BOLD signal of anterior insular cortex, using fMRI-BCI. During fMRI sessions of “training,” subjects were instructed to activate insula cortex, aided by visual contingent neurofeedback presented by means of a graphical thermometer that provided regular updated fMRI information of BOLD signal from right anterior insula (Figure 1). After few fMRI sessions, all participants were able to successfully regulate the BOLD signal in insula. Training resulted in a significantly increased activation cluster in the anterior portion of the right insula across sessions. As in other previous studies, self-regulation could not be achieved by a control group trained with sham feedback.

But, can learned insula self-regulation produce a measurable behavioral modification? In a subsequent study, we explored the relationship between brain self-regulation and emotional behavior (Caria et al., 2010). Healthy participants underwent four fMRI-BCI scanning sessions in order to train them to modulate the BOLD response in anterior insula guided by visual feedback (as in the previous study). This time, after each modulation block of self-regulation and baseline, participants were presented either with an emotionally negative or a neutral picture taken from IAPS. Immediately after presentation, participants were required to rate the picture using the Self-Assessment Manikin (Lang, 1980). As expected, participants learned to increase and decrease the BOLD response in insula guided by contingent feedback. Behavioral data showed a significant difference of valence ratings of the aversive pictures (emotionally negative) in the last session.

To confirm that learned self-regulation of insula could only be achieved by feedback of the BOLD signal of the ROI and not due to generalized brain activations, the feedback magnitude was computed by subtracting the BOLD signal of a reference slice (Caria et al., 2007, 2010). Hence, global activations in fact would decrease the magnitude of feedback. Furthermore, a control group of subjects was trained directly with information containing the average activity in a large brain area not encompassing left and right insula. This control group was unable to achieve insula self-regulation and did not show the behavioral modifications displayed by the experimental group.

These results demonstrate that fMRI-BCI manipulation of paralimbic regions such as insula is possible, and that self-regulation leads to modulations of the emotional response. Furthermore, these studies show that fMRI-BCI provides a novel approach in neuroscience for studying brain reorganization, in which the manipulation of the brain activity can be used as an independent variable that allows the study of subsequent behavioral modifications.

**Figure 2:**
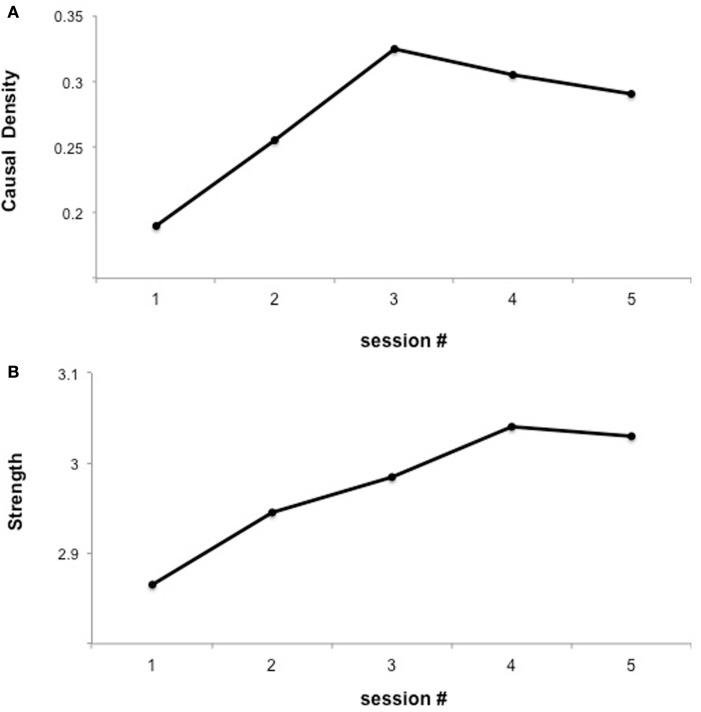
**“Functional interaction of brain regions of the emotional network across fMRI sessions of insula self-regulation.”** The figure shows the changes in causal density and connection strengths with feedback training. **(A)** Causal density in all the sessions. **(B)** Connection strength. The causal density of the functional network decreases in session 5 indicating substantial “pruning” [as shown in **(A)**] yet “strengthening” [as shown in **(B)**] of the connections between ROIs reproduced with permission from Lee et al. (2011).

In a subsequent analysis of the previous data, we explored whether learned self-regulated was associated with changes in brain network connectivity (Lee et al., 2011) using Granger causality modeling (GCM) (Seth, 2010), methodology that uses temporal information in one or more time-series of signals from a certain brain region to predict signal time courses in another, so that temporally directed influences rather than only correlations in activity between different brain areas can be generated (Seth, 2005, 2010; Abler et al., 2006). Connectivity analysis revealed an increase in the number of connections in the emotion network (including insula cortex, DLPFC, IFG, Putamen, ACC, and STG) followed by subsequent pruning of redundant connections and strengthening of potentially relevant connections (Figure 2). These results suggest that BCI could be used to build a more efficient neural pathway, which might be particularly relevant for conditions in which an abnormal neural connectivity is implied, as autistic disorders or schizophrenia.

The potential use of fMRI-BCI to modulate brain networks connectivity has also been confirmed in two recent studies in healthy populations. Hamilton et al. (2010) tested whether subjects can down regulate the activity of the subgenual anterior cingulate (sACC) cortex with fMRI-BCI, area that has been implicated in several mental disorders. Using “positive affect strategies” and visual contingent fMRI feedback, eight healthy women were able to down regulate the BOLD signal from sACC. The study included a PPI analysis of connectivity, which showed that BCI training was associated with a decreased correlation (connectivity) between sACC and posterior cingulate cortex. On their part, Zotev et al. (2011) trained individuals to self-regulate the BOLD signal of left amygdala by real-time fMRI. Interestingly, the functional connectivity of the network including frontopolar, ACC, and DLPFC with amygdala cortex increased significantly across the fMRI runs.

### fMRI-BCI in schizophrenia

Particularly in schizophrenia, fMRI-BCI could offer a new method to directly correct brain dysfunctions that accounts for the stable and intractable symptoms and cognitive deficits of the disorder.

**Figure 3:**
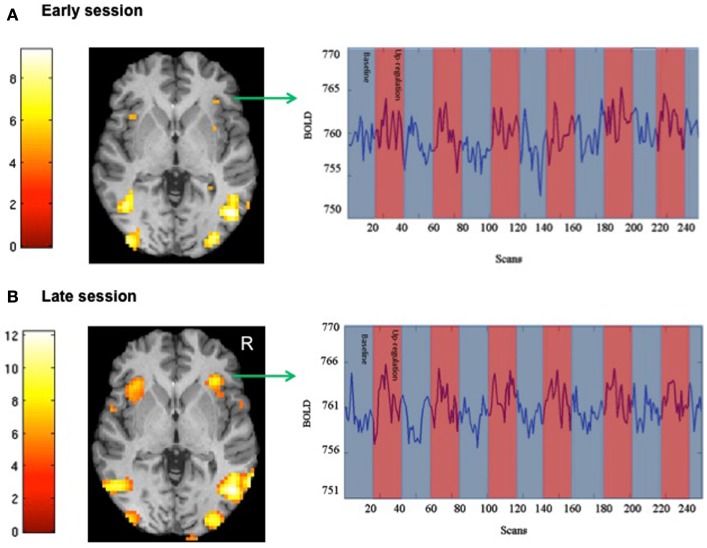
**“SPM analysis and BOLD time-series of a representative subject.”** The figure shows the cluster activations for the contrast self-regulation vs. baseline during **(A)** an early and **(B)** a late session of fMRI-BCI training [*P* < 0.05 (FWE), coordinate *z* = 0]. BOLD time-series for right anterior insula are also shown in the respective sessions (blue and red bars represent the experimental conditions baseline and self-regulation, respectively) adapted with permission from Ruiz et al. (2013).

**Figure 4:**
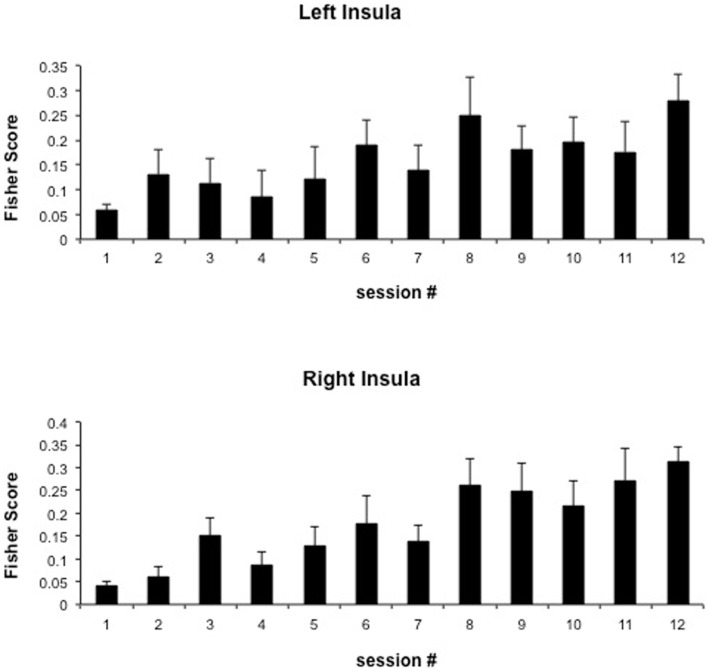
**“Group analysis BOLD change across training.”** Bars represent insula self-regulation learning across 12 sessions of fMRI-BCI, measured by the Fisher score (and standard error), of 9 schizophrenia patients. Fisher score is a measure of signal change that considers both the variance and the mean of the BOLD signal values extracted from the ROIs adapted with permission from Ruiz et al. (2013).

**Figure 5:**
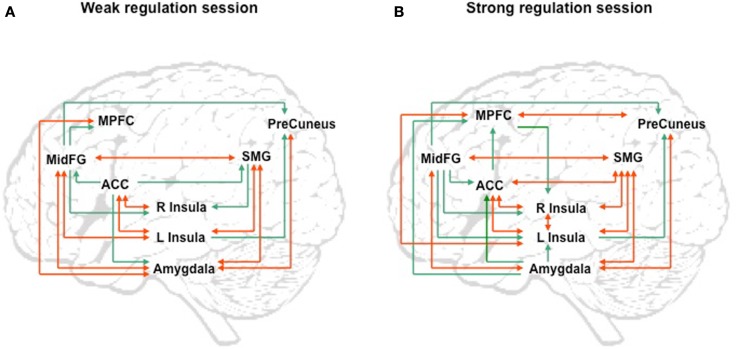
**“Group analysis of effective connectivity changes due to fMRI-BCI training using Granger causality modeling (GCM).” (A)** Direct influences of the emotional network during self-regulation in the weakest regulation session. **(B)** Direct influences of the emotional network during self-regulation in the strongest regulation session. Red arrows indicate bidirectional influences between ROIs. MidFG: left middle frontal gyrus; MPFC: left medial prefrontal cortex; ACC: left anterior cingulate cortex; L Insula: left anterior insula; R Insula: right anterior insula; SMG: left supra marginal gyrus adapted with permission from Ruiz et al. (2013).

**Figure 6:**
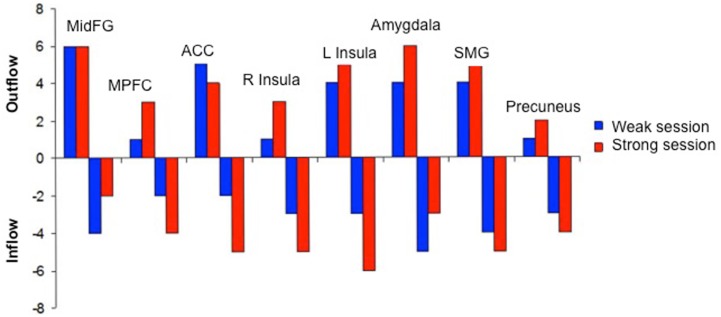
**“Outgoing and incoming connections of each ROI of the emotional network during up-regulation.”** Outflow: number of outgoing connections from each of the ROIs. Inflow: number of incoming connections to each of the ROIs. The figure graphically represents the connections displayed in Figure 5. MidFG, left middle frontal gyrus; MPFC, left medial prefrontal cortex; ACC, left anterior cingulate cortex; L Insula, left anterior insula; R Insula, right anterior insula; SMG, left supra marginal gyrus adapted with permission from Ruiz et al. (2013).

We attempted for the first time to apply fMRI-BCI on schizophrenic patients (Ruiz et al., 2013). The first aim of this study was to evaluate if schizophrenic subjects can achieve volitional regulation of anterior insula cortex by fMRI-BCI training, and to explore the relationship between the capability to self-regulate and other aspects of the symptomatology. Insula cortex was chosen as the region of interest based on the increasing evidence suggesting that insula dysfunction might be critically involved in different aspects of the schizophrenia psychopathology (Crespo-Facorro et al., 2000, 2001; Paradiso et al., 2003; Yoo et al., 2005; Makris et al., 2006; Saze et al., 2007; Meisenzahl et al., 2008; Price et al., 2010). Nine chronic schizophrenia patients moderately symptomatic and under antipsychotic medication were recruited for a long-training regime consisting of 12 sessions of fMRI-BCI, in which patients learned to self-regulate anterior insula by online visual feedback. Our results showed that after training, patients were able to learn to self-regulate the BOLD response in insula cortex. A group linear regression analysis performed across all sessions of training showed a significant increase in BOLD signal for both left and right anterior insula (Figures 3 and 4). Interestingly, the capability to self-regulate was negatively correlated with the severity of negative symptoms and the duration of the illness, suggesting that factors interfering with self-regulation may start in early stages of the disease and develop rapidly thereafter. Secondly, we explored whether self-regulation was associated with a behavioral modification over face emotion recognition. After each block of insula self-regulation and baseline, patients were presented emotional faces displaying disgust and happy expressions, and asked to recognize the emotions. Following learned self-regulation of insula, patients detected significantly more disgust faces, in line with the extensive evidence of the role of insula cortex in the recognition disgust faces (Phillips et al., 1997, 2004; Anderson et al., 2003; Schroeder et al., 2004). Patients detected less happy faces during self-regulation, which may lead one to speculate that heightened activations of anterior insula might tilt the balance from positive valance stimuli (in this case happy emotional faces), toward a more negative valence. Finally, we explored whether learned brain regulation leads to modifications in the effective connectivity of the emotional network, using GCM. Effective connections among insula cortex, amygdala, and MPFC were enhanced at the end of the training. In addition, the density of the network as a whole was enhanced. This change was reflected in the larger causal density (CD: the fraction of interactions among ROIs in a network that are causally significant) of the network in the strongest session of regulation compared with the weakest session of regulation (Figures 5 and 6).

These results demonstrated that with sufficient training, schizophrenia patients are able to learn volitional regulation of the insula cortex by fMRI-BCI. Particularly for schizophrenia, it has not been previously shown that patients would be able to self-induce neural activation modifications, leading to behavioral effects. Here we could demonstrate for the first time a completely novel approach in psychiatric disorders, involving functional brain changes due to self-regulation of cerebral activation patterns. Learned self-regulation led to changes in the perception of emotional faces, a major dysfunction in schizophrenia, showing that behavioral modulation by this new technique in schizophrenia is possible. Finally, the enhancement of the connections in brain emotional network suggests that fMRI-BCI can be used to “re-connect” the schizophrenic abnormal neural connectivity.

### Direct modulation of brain connectivity with fMRI-BCI

So far, the few studies using fMRI-BCI that have analyzed brain connectivity suggest that reorganization or the functional connection in the brain is possible. However, at the neural level, enhancements of brain connections have been achieved as a positive by-product of single ROI self-regulation training, and not by a “direct” training of the brain connectivity. Progress in real-time fMRI methodology now enables the direct feedback of the connections between brain areas, creating exciting possibilities for the direct modulation of brain networks by self-regulation.

In a recent study, we attempted to use fMRI-BCI to “directly” enhance neural connectivity in a group of young healthy subjects (Ruiz et al., 2011). Participants were trained to increase the functional connectivity between fronto-temporal cortex during fMRI-BCI training, by online visual contingent feedback.

The equation to compute the magnitude of feedback was:

$$\begin{array}{rcll} \text{Magnitude of feedback}= & \left(\text{BOLD ROI 1}+\text{BOLD ROI2}\right) & & \\ & \times \left(\text{1}+\text{EC}\right) & & \end{array}$$

where, ROI 1 was inferior frontal gyrus, ROI 2 was the posterior part of STG, and EC was the correlation coefficient derived from the BOLD time-series in these two ROIs.

**Figure 7:**
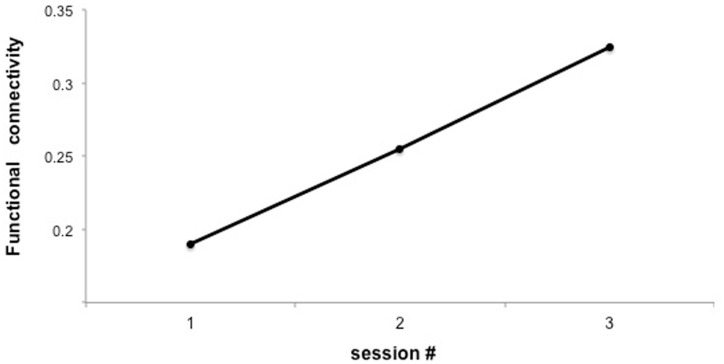
**“Functional connectivity across three sessions of training in a representative subject.”** *Y*-axis represents the *F*-value for the connection between IFG and posterior STG under the autoregressive model, based on Granger causality analysis.

**Figure 8:**
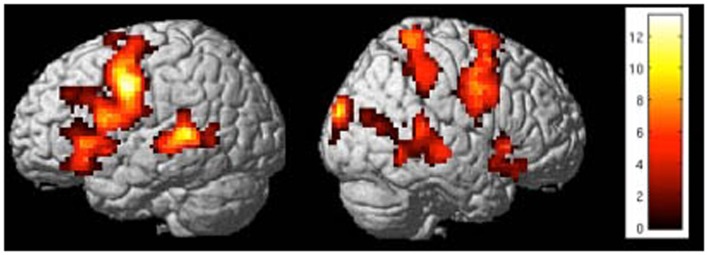
**“Activation clusters for the contrast up-regulation vs. baseline during the last session of self-regulation training in a representative subject.”** Important brain activations can be seen in posterior STG and frontal areas (included IFG) in the left hemisphere [*p* < 0.05 (FWE), extent threshold *k* = 20].

Preliminary results suggested that participants were able to learn self-regulation of the connectivity between IFG and STG (Figures 7 and 8). Secondly, we explored the behavioral modification induced by BCI training in an automatic semantic priming task, a paradigm associated with the activation of fronto-temporal areas (Sass et al., 2009). An enhancement of the priming effect (shorter reaction times) was observed following self-regulation of the fronto-temporal coupling. Albeit preliminary, these results suggest that it is possible to directly enhance brain network connectivity with fMRI-BCI training, leading to measurable behavioral modifications.

## Conclusion

Growing evidence has shown that an abnormal connectivity of different brain areas is central to schizophrenia. Considering that no therapeutic approach has been designed to alleviate this problem, fMRI-BCI offers a novel and promising possibility to enhance brain connectivity, without adverse effects. Schizophrenia patients are able to learn self-regulation of single brain areas using this technology, leading to behavioral modifications and brain network modulation. Furthermore, the possibility to train patients to directly enhance brain connectivity offers new and exciting possibilities for brain network remodeling.

As fMRI-BCI is an emerging methodology, there are still unanswered questions regarding technical aspects and clinical applications. As an example, although much is known regarding how BOLD changes are associated with underlying neural activity, the fact that both neural excitatory and inhibitory responses lead to increase in BOLD (Logothetis, 2008), makes it impossible to say which of these two phenomena is associated with self-regulation of BOLD or network connectivity. Whether learned self-regulation leads to neural changes associated with learning (e.g., pruning, consolidation, or strengthening of the used connections and networks) has not been yet explored either.

Furthermore, although fMRI-BCI training purportedly implies learning through operant conditioning, little is known of the deeper mechanism of learning using this methodology. As an example, Ruiz et al. (2013), schizophrenia patients reported the use of cognitive strategies for regulation at the beginning of the experiment. However, at final stages of the training some of them reported that the control of the feedback was achieved with less effort or even “*automatically*” or “*unconsciously*.” In that sense, future research must improve our understanding regarding the processes by which subjects achieve brain self-regulation with fMRI-BCI, including the degree to which explicit or implicit mechanisms are used.

Regarding clinical applications, important questions need to be addressed before the use of this methodology as a therapeutic tool becomes a reality. In fact, very few studies have explored whether brain self-regulation persists if feedback is removed, with dissimilar results (Sitaram et al., 2012; Ruiz et al., 2013). Similarly, only few studies have assessed whether the capability for self-regulation will persist in the following days after the rtfMRI-BCI training (Yoo et al., 2008; Haller et al., 2010), and no study has analyzed these aspects in a clinically significant long-term.

Despite these current limitations, we believe that the presented results open the door for future applications of fMRI-BCI as a research tool and therapeutic approach against brain diseases. In addition, it can also serve as a way for stimulating self-efficacy in patients and offers the possibility to remodel once own brain.

Recent progress in real-time pattern classification and feedback of fMRI signals (LaConte et al., 2007; Sitaram et al., 2011) would enable feedback of spatial and temporal patterns of connectivity among a number of brain regions in a network (e.g., emotion network), instead of just two or three regions. Such a system is being developed in our laboratory to train patients to mimic or emulate brain network connectivity of healthy individuals with contingent feedback. Future studies will explore the potential applications of this new technique in psychiatric disorders.

## Conflict of Interest Statement

The authors declare that the research was conducted in the absence of any commercial or financial relationships that could be construed as a potential conflict of interest.
